# Antibody–Ferrocene Conjugates as a Platform for Electro-Chemical Detection of Low-Density Lipoprotein

**DOI:** 10.3390/molecules27175492

**Published:** 2022-08-26

**Authors:** Daria Rudewicz-Kowalczyk, Iwona Grabowska

**Affiliations:** Institute of Animal Reproduction and Food Research, Polish Academy of Sciences, Tuwima 10, 10-748 Olsztyn, Poland

**Keywords:** low-density lipoprotein (LDL), immunosensor, ferrocene–antibody conjugates, electrochemical detection

## Abstract

Low-density lipoprotein (LDL) is a cardiac biomarker identified in the pathology of cardiovascular disease (CVD). Typically, the level of LDL is calculated using the Friedewald relationship based on measured values of total cholesterol, high-density lipoproteins (HDL), and triglycerides. Unfortunately, this approach leads to some errors in calculation. Therefore, direct methods that can be used for fast and accurate detection of LDL are needed. The purpose of this study was to develop an electrochemical platform for the detection of LDL based on an antibody–ferrocene conjugate. An anti-apolipoprotein B-100 antibody labeled with ferrocene was covalently immobilized on the layer of 4-aminothiophenol (4-ATP) on the surface of gold electrodes. Upon interaction between LDL and the antibody–ferrocene conjugate, a decrease in the ferrocene redox signal registered by square wave voltammetry was observed, which depends linearly on the concentration from 0.01 ng/mL to 1.0 ng/mL. The obtained limit of detection was equal to 0.53 ng/mL. Moreover, the satisfied selectivity toward human serum albumin (HSA), HDL, and malondialdehyde-modified low-density lipoprotein (MDA-LDL) was observed. In addition, the acceptable recovery rates of LDL in human serum samples indicate the possible application of immunosensors presented in clinical diagnostics.

## 1. Introduction

The European Atherosclerosis Society Consensus Panel has reported that low-density lipoprotein (LDL) is a casual factor in the pathology of atherosclerosis cardiovascular disease (ASCVD) [1]. Besides the physiological function of LDL particles as a transporter of cholesterol to cells in the body, their elevated level—and hence the possible modifications, including, for example, oxidation—can lead to LDL accumulation in the arterial wall and ASCVD development [2]. It is estimated that the intensive reduction in LDL to a level less than 100 mg/dL has a positive effect on lowering the risk of cardiovascular diseases. However, according to the guidelines of the European Society of Cardiology (ESC) and the European Atherosclerosis Society (EAS), the cut-off value of LDL that is used to define a high risk of ASCVD in clinical trials is >190 mg/dL [3,4]. Therefore, regularly testing the LDL level is of particular importance. In clinical practice, the LDL particle level is estimated using the standard lipid panel, including total cholesterol (TC), high-density lipoprotein (HDL), and triglycerides (TGs) by the Friedewald equation: LDL = TC-HDL-TGs/5. However, this formula cannot be accurate if the TG level is higher than 400 mg/dL [5]. Thus, there is a growing need for the development of direct LDL measurement methods that are robust and easy to use.

Electrochemical immunosensors have tremendous potential to be an attractive alternative to traditional methods, mainly because of their high selectivity and sensitivity, the simple construction of measuring devices and their operation, and the possibility of application in medical diagnostics at the point of care [6,7]. The principle of electrochemical immunosensors is based on the recognition process between bioreceptors—an antibody (Ab) and an antigen (Ag). This action is next converted into analytically useful signals by means of an electrochemical transducer [8]. The Ab–Ag immunocomplex formation can be observed by changes in the redox activity of different labels, including ferrocene, thionine, methylene blue, hemin, or antraquinone [9], and gold, silver, or quantum dots attached to antibodies [10,11]. There are possibilities for covalent conjugation of electrochemical labels on antibodies by combining amino groups of labels with carboxyl groups of antibodies [12]. The antibody–electroactive label conjugates could be used in immunosensor construction in sandwich or direct signal format. In the case of sandwich format, there are two type of antibodies: unlabeled primary capture antibodies and redox-active labeled signaling secondary antibodies. In this case, upon sandwich complex formation, an increase in peak current is observed [13]. In the direct signal format, the creation of an immunocomplex causes a decrease in the electrochemical signal of the redox-active label attached to antibodies due to the spatial blocking of the electrode surface [14].

In this work, we concentrated on the use of antibody–ferrocene conjugates in the construction of a direct signal immunosensor for LDL detection. Here, the antibody–redox-active molecule conjugates act simultaneously as capture antibodies as well as signaling antibodies. Therefore, there is no need for the addition of a redox marker into the supporting solution. Among many literature examples of electrochemical immunosensors for the detection of LDL, those including the use of external markers in the solution are the most popular [15,16,17,18,19,20]. There are few examples of electrochemical immunosensors for LDL detection which do not require an external redox marker. One of the recent examples presents a detection method of LDL using a non-faradaic impedimetric immunosensor [21]. Other papers [22,23] show the utilization of NiO thin film wherein the changes in the oxidation process of the Ni(II) state of NiO into the Ni(III) state of NiOOH were used for the detection of LDL upon the antigen–antibody immunocomplex formation.

The purpose of this work was to apply anti-LDL monoclonal antibodies conjugated with electroactive ferrocene (AbM-anti-apoB-Fc) in the construction of an electrochemical platform for the sensing of LDL in serum without the need for an external redox-active molecule in the supporting solution. The procedure of antibody conjugation with amine-reactive N-hydroxysuccinimide (NHS) ferrocene was carried out on high-capacity magnetic Protein A beads [24,25]. Antibodies were obtained that maintain their functionality, and the procedure of their labeling has many advantages over the solution-based methods, including, among others, rapid processing, simple washing and incubation steps, the possibility of labeling samples with low amounts of antibodies, and efficient recovery. In this paper, NH_2_ groups of 4-aminothiophenol, previously self-assembled on a gold electrode surface, were used for covalent linking of AbM-anti-apoB-Fc. The detection of LDL was performed by square wave voltammetry, registered as a decrease in the electrochemical response of ferrocene upon interfacial immunoreaction between surface-immobilized antibody–ferrocene conjugates and LDL present in the solution. The proposed strategy allowed us to achieve a detection limit for LDL of about 0.53 ng/mL and satisfying recovery rates in human serum.

## 2. Results and Discussion

### 2.1. On-Bead Antibody Conjugation Using Amine-Reactive Ferrocene (NHS-Ferrocene)

Immunoglobulins (IgGs) are the most frequently used bioreceptors in electrochemical immunosensors. These molecules consist of about 20 NH_2_ groups derived from lysines, being attractive sites for conjugation. These groups are distributed irregularly across the whole molecule and are characterized by solvent accessibility capable of forming amide bonds with carboxylic groups [26]. Two methods of antibody conjugation in solution with ferrocene derivatives have been reported thus far [27]. The first is attaching ferrocenecarboaldehyde (Fc-CHO) to IgG via the Schiff base and reduction by sodium borohydride, and the second involves attaching an amine-reactive sulfo-NHS ester (N-hydroxysulfosuccinimide) of ferrocene monocarboxylic acid to IgG. These conjugations have been carried out in solution involving multistep reactions, several incubations, and buffer exchange. Moreover, these processes require relatively high concentrations of antibodies, above 1 mg/mL. To overcome these limitations, solid-phase labeling methods have been developed [28,29], further extended to on-bead labeling methods [24,25].

In this work, we applied an on-bead conjugation method to apolipoprotein B monoclonal antibody (AbM-anti-apoB) and ferrocene carboxylic N-hydroxysuccinimide ester (Fc-NHS) in order to obtain conjugates characterized by appropriate functionality.

**Scheme 1:**
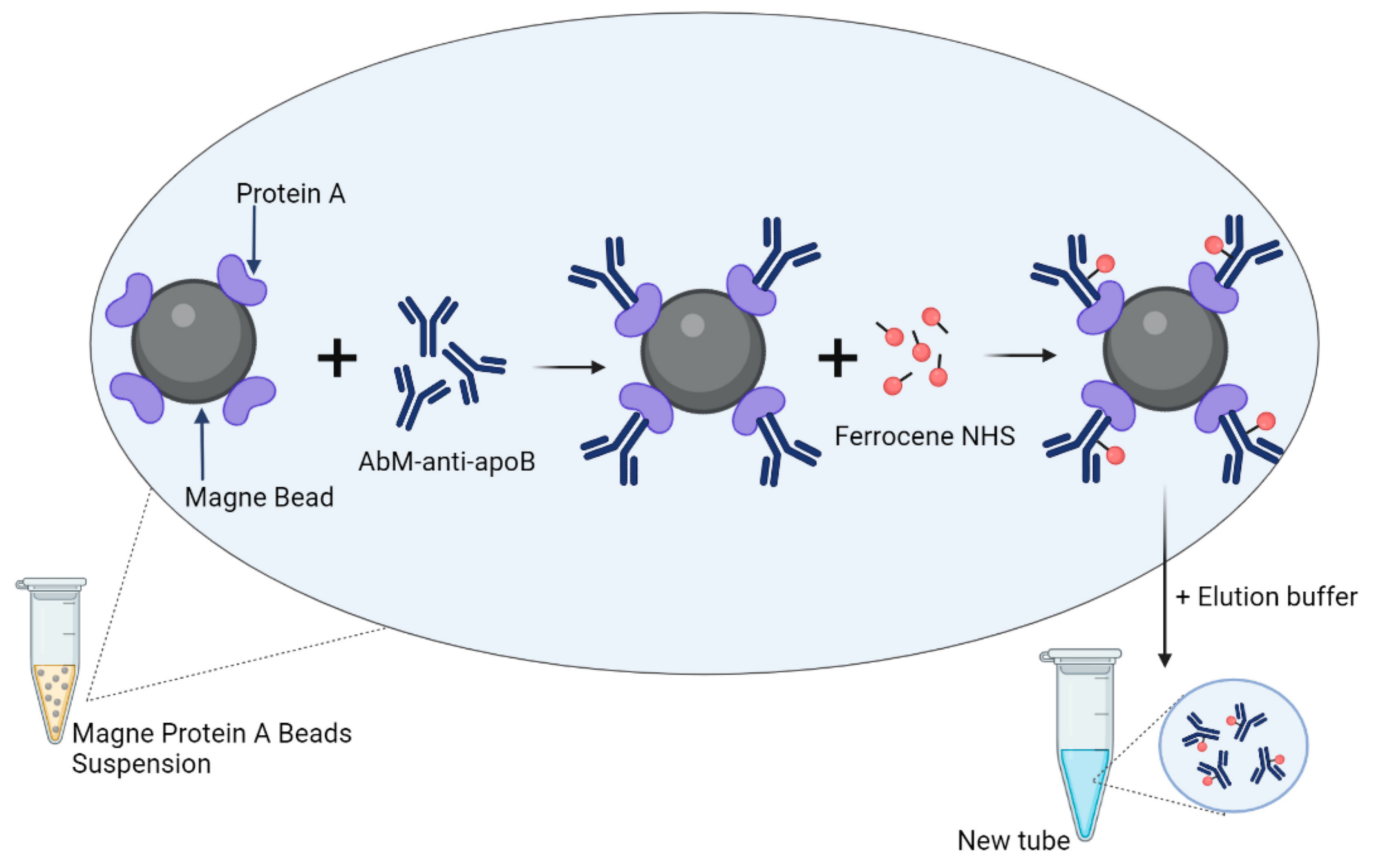
Schematic illustration of the conjugation procedure between the AbM-anti-apoB and ferrocene carboxylic N-hydroxysuccinimide ester (ferrocene-NHS).

The schematic illustration of the conjugation procedure is presented in Scheme 1, and the procedure is described in Section 4. In brief, high-capacity magnetic Protein A beads are used for capturing AbM-anti-apoB in the first step, followed by conjugation with ferrocene derivative containing NHS ester. The final step involves the elution of conjugated antibodies from the beads. Conjugated antibodies are then ready for application in electrochemical immunosensor construction.

### 2.2. Preparation of Immunosensor Platform Based on Antibody–Ferrocene Conjugates

Deposition of antibodies onto the sensor surface in an oriented way that allows the preservation of their biological activity is essential for proper sensor performance. Many controllable and site-specific immunoglobulin deposition strategies on surfaces have been recently reported, including the examples for two-step immobilization mediated by the linkers protein A or G, Fc-binding domain, biotin(strept)avidin, SpyTag/SpyCatcher, and others [30].

In this work, we applied 4-aminothiophenol (4-ATP) as a linker possessing a NH_2_ group at the end for covalent immobilization of antibody–ferrocene conjugates. Recently, we have shown that 4-ATP immobilized on the surface of gold delivers NH_2_ groups, which are beneficial for covalent immobilization of antibodies [31]. Other authors have also confirmed these phenomena [32,33,34].

**Scheme 2:**
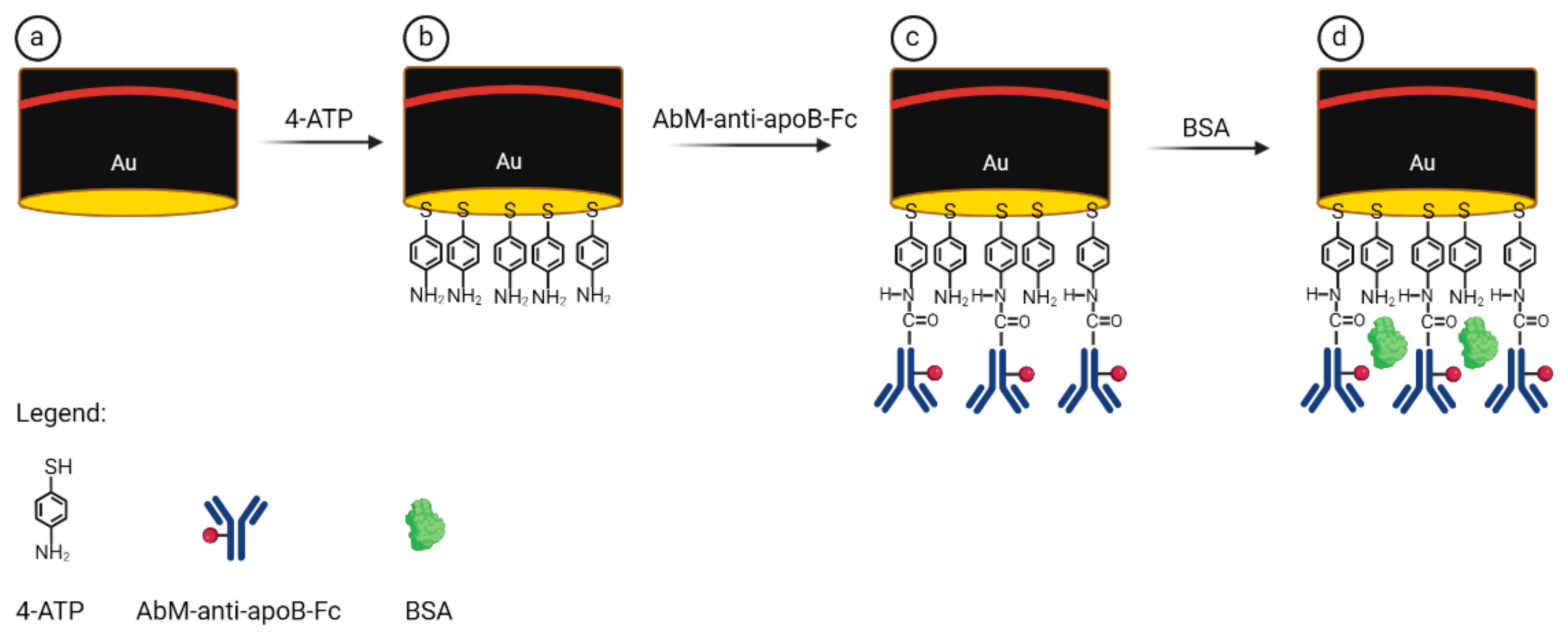
Schematic representation of the electrochemical immunosensor preparation steps: (**a**) bare Au, (**b**) Au/4-ATP, (**c**) Au/4-ATP/AbM-anti-apoB-Fc, (**d**) Au/4ATP/AbM-anti-apoB-Fc/BSA.

The procedure of immunosensor preparation is presented in Scheme 2. Thus, upon covalent immobilization of 4-ATP (step b), the AbM-anti-apoB-Fc conjugates, activated by EDC/NHS, were covalently attached to amine groups present on the gold electrode surface (step c). The last step involved coating the free places on the electrode surface with BSA molecules in order to eliminate non-specific interactions (step d).

### 2.3. Electrochemical Characterization of Antibody–Ferrocene Conjugates Deposited on Gold Surface

**Figure 1:**
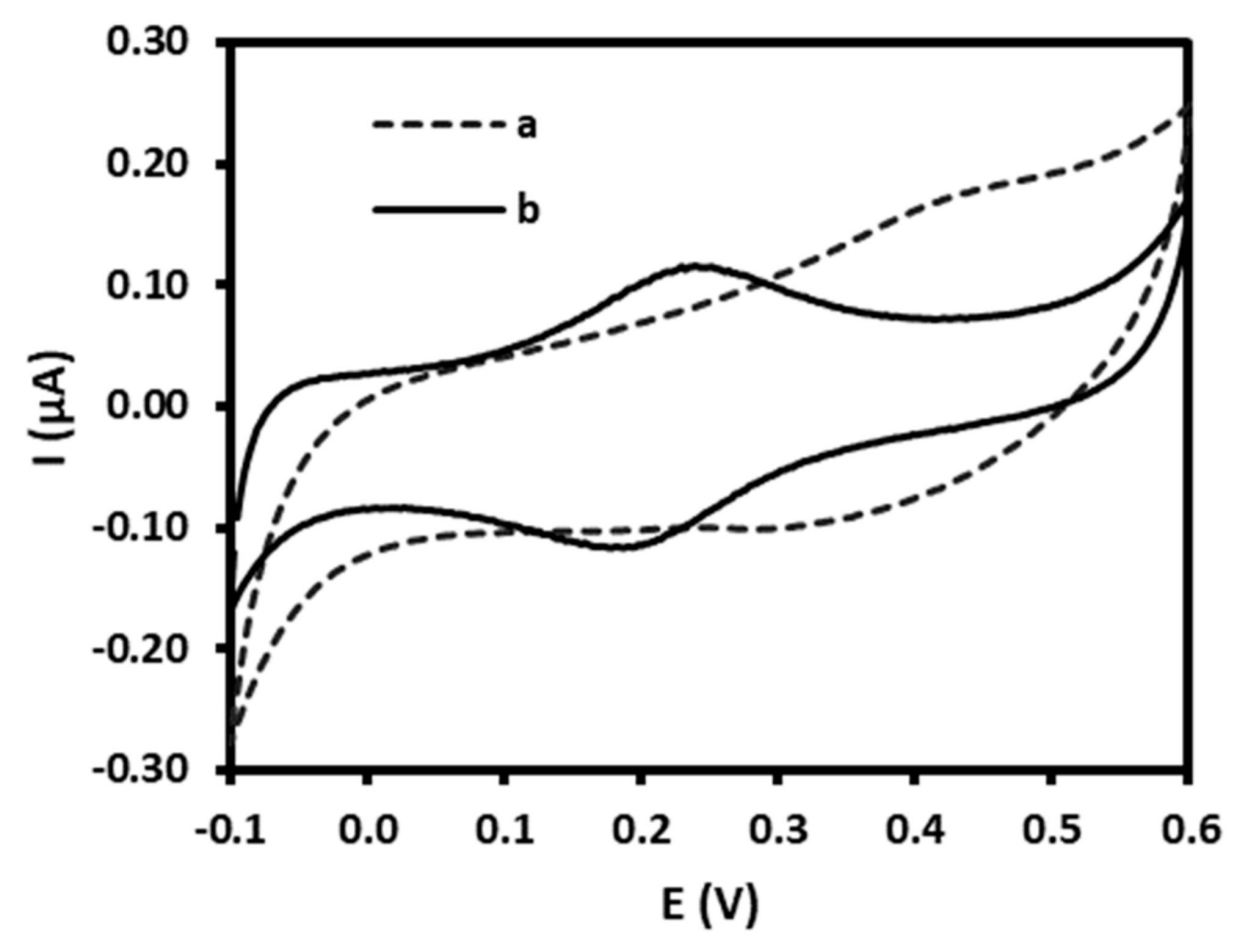
Cyclic voltammograms curves registered for (**a**) bare gold electrode surface and (**b**) immunosensor at the final step of modification: Au/4-ATP/AbM-anti-ApoB-Fc/BSA. Supporting electrolyte: 0.1 M sodium phosphate buffer, 0.15 M Na_2_SO_4_, pH 7.0. Scan rate: 0.1 V/s.

Ferrocene (Fc) is an organometallic sandwich complex consisting of a central iron atom located between two cyclopentadienyl rings (Fc: Fe^II^(C_2_H_5_)_2_). This complex can be easily electrochemically oxidized, resulting in its oxidized form, ferrocenium cation (Fc^+^: [Fe^III^(C_2_H_5_)_2_]^+^). The process is highly reversible [35]. In order to confirm the presence of ferrocene-tagged antibodies on the surface of gold electrodes, cyclic voltammetry measurements were recorded for the bare gold electrode and upon the antibody immobilization in buffer solution containing 0.1 M sodium phosphate buffer, 0.15 M Na_2_SO_4_, pH 7.0. Figure 1 exhibits the cyclic voltammograms of gold electrodes (curve a) and after coating with antibody–ferrocene conjugates (curve b) performed at a scan rate of 0.1 V/s. Immobilization of these conjugates on the surface of the gold electrodes results in the appearance of two peaks, the first at the position of 0.239 ± 0.005 V, which is related to the oxidation reaction of Fe^II^(C_2_H_5_)_2_ -1e^−^ → [Fe^III^(C_2_H_5_)_2_]^+^, and the second at the position of 0.195 ± 0.008 V (n = 6), which is characteristic of the reduction process [Fe^III^(C_2_H_5_)_2_]^+^ +1e^−^ → Fe^II^(C_2_H_5_)_2_ [36].

**Figure 2:**
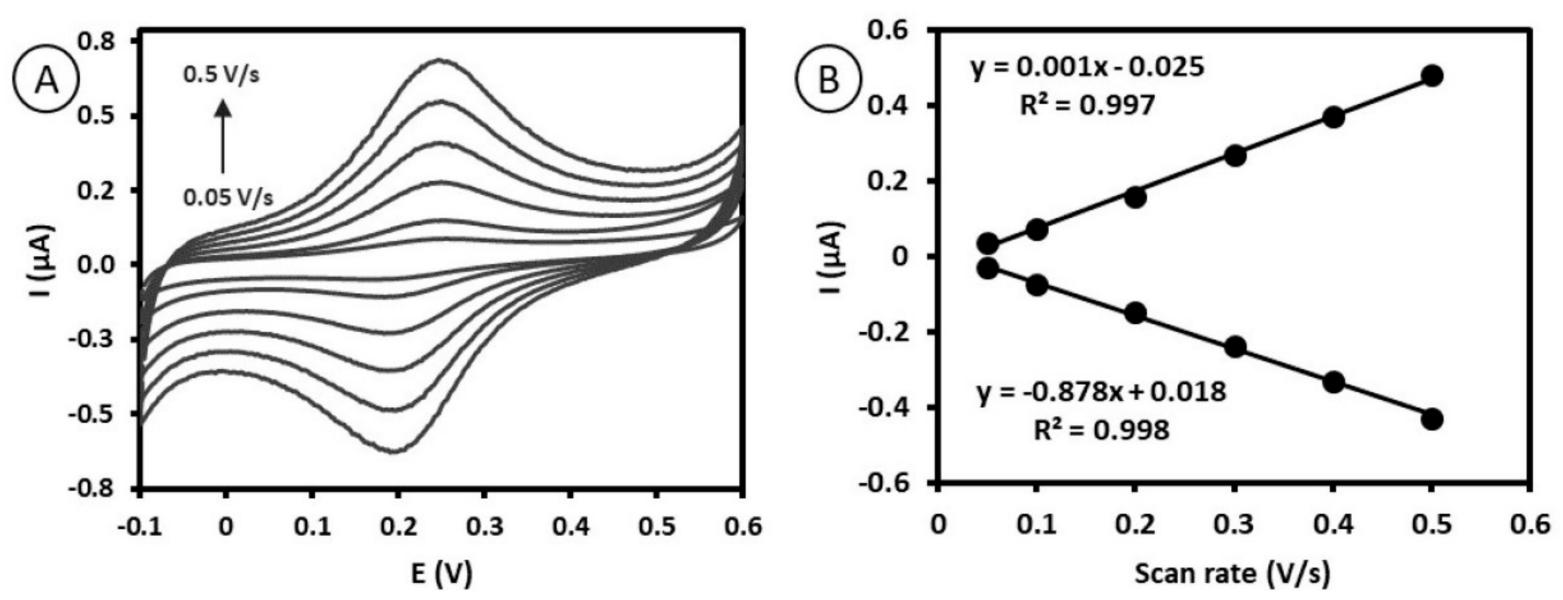
(**A**) Cyclic voltammograms of the immunosensor Au/4-ATP/AbM-anti-ApoB-Fc/BSA recorded at various scan rates: 0.05, 0.1, 0.20, 0.3, 0.4, 0.5 V/s. Supporting electrolyte: 0.1 M sodium phosphate buffer + 0.15 M Na_2_SO_4_, pH 7.0. (**B**) Plots of the oxidation and reduction peak current of ferrocene attached to antibodies vs. scan rates.

To further confirm the surface-confined nature of ferrocene, the anodic and cathodic peak currents with changing scan speeds (from 0.05 to 0.5 V/s) were registered (Figure 2A). As presented in Figure 2B, the relationship between anodic and cathodic peak current values vs. scan rates was linear between 0.05 and 0.5 V/s, indicating that the process is not diffusion-dependent and confirming the location of AbM-anti-apoB-Fc on the surface of the gold electrodes [37]. The surface coverage was determined from the slope of the relationship between I_p_ and ν according to the following equation [37]:

(1) $$I_{p}=\frac{n^{2}\cdot F^{2}\cdot \Gamma \cdot A\cdot \nu }{4\cdot R\cdot T}$$

where I_p_ is the anodic peak current, Γ is the surface coverage (mol/cm^2^), n is the number of electrons transferred, F is the Faraday constant (96485 C/mol), A is the surface area of the electrode (0.02 cm^2^), ν is the scan rate (0.1 V/s), R is the gas constant (8.314 J/mol·K), and T is temperature (298.15 K).

The calculated surface coverage of ferrocene assembled on the gold electrode surface was equal to 4.4 ± 0.8 × 10^−11^ mol/cm^2^. The lack of literature data for similar systems makes it impossible to compare this value with other works.

### 2.4. Quantitative Electrochemical Detection of LDL by Immunosensor Based on Antibody–Ferrocene Conjugates

The main purpose of this work was to develop a direct electrochemical immunosensor for the detection of LDL. Here, ferrocene molecules conjugated to antibodies which are attached to the surface of gold electrodes undergo a redox reaction. During the experiments presented in this study, we used square wave voltammetry to monitor direct oxidation/reduction of ferrocene before and after the formation of a complex between AbM-anti-apoB-Fc and LDL. The immunocomplex creation leads to the spatial blocking of the electrode surface, which affects the measured oxidation/reduction current. As expected and supported by other authors [14,36], we observed a decrease in current value, which is related to the concentration of LDL in the sample.

**Figure 3:**
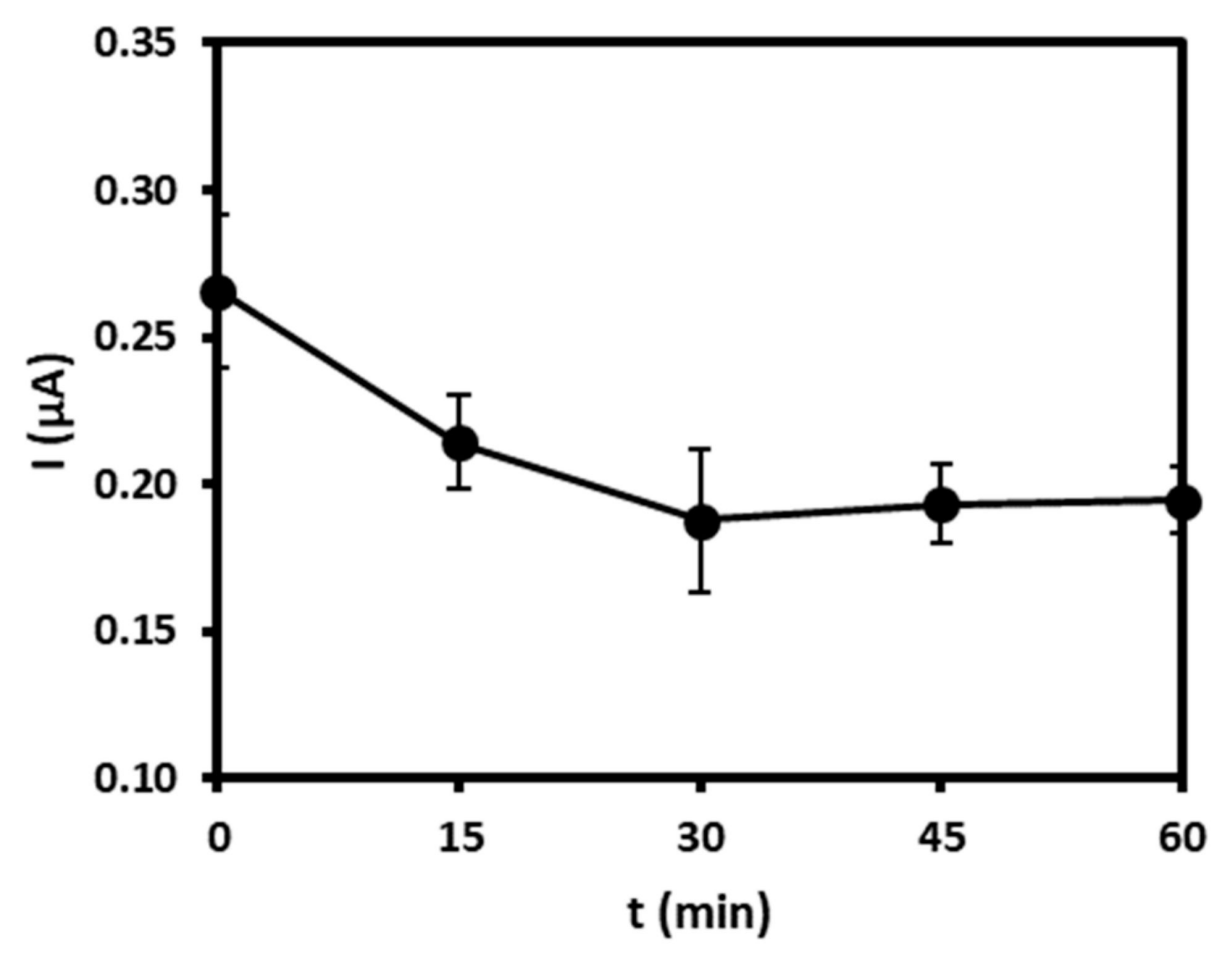
Optimization of the incubation time (15, 30, 45, 60 min) of LDL solution of 0.5 mg/mL on AbM-anti-apoB-Fc-modified gold electrodes registered using square wave voltammograms in buffer (pH = 7.0).

The performance of the immunosensor is closely related to the various parameters of its preparation procedures. Therefore, we optimized in the first experiment the incubation time of the antigen (LDL) on the surface-confined antibody to obtain a stable signal of ferrocene current. For this purpose, AbM-anti-apoB-Fc-modified electrodes were incubated with LDL solution of 0.5 mg/mL for different time sets, 15, 30, 45, and 60 min, and square wave voltammograms were registered for each time. As can be seen in Figure 3, the time of 30 min is efficient to read the stable decrease in the ferrocene current, resulting from the complete saturation of the antibody with the antigen. Therefore, a 30 min incubation time was maintained in the following experiments.

**Figure 4:**
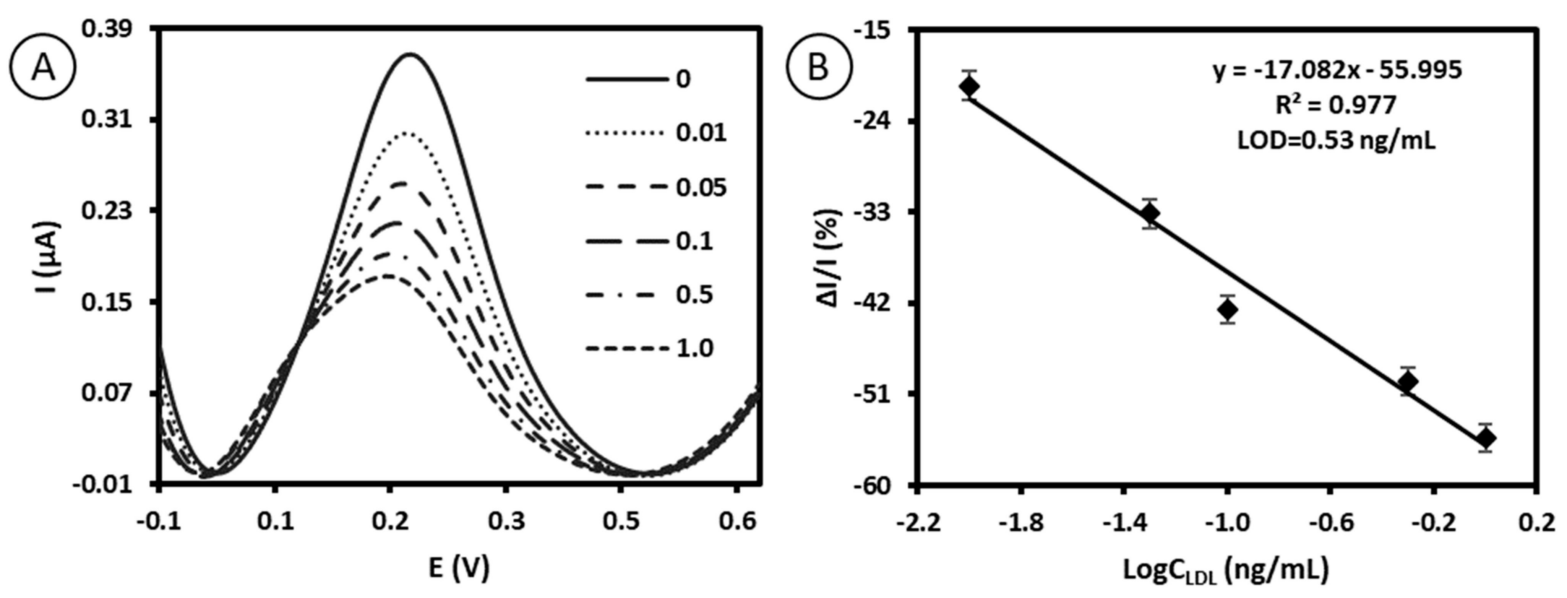
(**A**) Square wave voltammograms recorded for immunosensor upon contact with LDL standard solution with different concentrations (0, 0.01, 0.05, 0.1, 0.5, 1.0 ng/mL). The measuring parameters of SWV: step potential = 5 mV; square wave frequency = 10 Hz; amplitude = 50 mV. (**B**) The relationship between relative intensity of ferrocene redox current vs. log concentration of LDL (ΔI = I_n_ – I, where I_n_ is the peak current value registered at particular “n” concentrations of LDL and I is the peak current value measured in buffer free of LDL (n = 5)). Measuring conditions: 0.1 M sodium phosphate buffer, 0.15 M Na_2_SO_4_, pH 7.0.

The immunosensor in the last step of preparation, Au/4-ATP/AbM-anti-ApoB-Fc/BSA, was characterized by square wave voltammetry in buffer (0.1 M sodium phosphate buffer, 0.15 M Na_2_SO_4_, pH 7), in the absence of LDL. We observed a peak current in the range of 0.34 ± 0.03 µA and at the position of 220.7 ± 2.1 mV (n = 5) derived from the oxidation/reduction of ferrocene conjugated to antibodies. Subsequently, the electrodes were stimulated with 10 µL solution of LDL at the particular concentrations, and a decrease in the maximum peak current was observed in the range from 0.01 ng/mL to 1.0 ng/mL of LDL, as presented in the Figure 4A. As the concentration of LDL increases, the amount of immunocomplexes between AbM-anti-ApoB-Fc and LDL increases. Due to this spatial blocking, the “local” environment of the ferrocene species is altered, and as a consequence, a decrease in the signal intensity of the Fc/Fc^+^ current is observed [14,36]. The relative intensity of the redox Fc/Fc^+^ current vs. log of the concentration of LDL follows a linear trend from 0.01 ng/mL to 1.0 ng/mL with regression equations ΔI/I = −17.082, logC_LDL_ − 55.995, and a correlation coefficient R^2^ of 0.977. We tested the concentration range in which the linear trend between the relative intensity of redox Fc/Fc+ current vs. log of concentration of LDL has been observed (see Figure 4B). The concentration range tested in this work is far from the critical concentration of LDL for monitoring ASCVD in clinical applications > 190 mg/dL. Our method offers ultra-high sensitivity, which is an advantage of electrochemical immunosensors. Therefore, in this case, it is possible to dilute the sample containing LDL of approx. 190 mg/dL about 1 × 10^7^ times to reach the tested range of 0.5 ng/mL LDL and thus reduce the biological matrix effect (the possible interferences for components of serum).

The detection limit (LOD) was calculated according to the following equation [38]:

(2) $$\mathrm{LOD}=\frac{3.3\sigma }{S}$$

where σ is the standard deviation of the response and S is the slope of the calibration curve.

The calculated detection limit was equal to 0.53 ng/mL.

The comparison of an immunosensor based on antibody–ferrocene conjugates for the detection of LDL presented in this work with other immunosensors already reported (Table 1) highlights its advantages. First of all, most examples [15,16,17,18,19] concern immunosensors in which detection requires the use of redox probes in the solution, e.g., ferri/ferrocyanide ([Fe(CN)_6_]^3−/4−^, which in fact could cause the denaturation of some proteins [39]. In addition, this approach is not appropriate for point-of-care systems, even if the given immunosensor has a better detection limit [15]. Moreover, two existing examples presenting the use of nanostructured NiO as a matrix for an electrochemical immunosensor, which eliminates the toxic mediator in the solution, are characterized by much worse detection limits than the immunosensor presented in this work [22,23]. In general, point-of-care tests should be easy to use, based on the analysis of a single drop of blood or saliva. As proposed in this work, antibody–ferrocene conjugates deposited onto the surface of gold electrodes can offer an attractive alternative for point-of-care detection of LDL because in this system, there is no need for an additional external redox marker to be added to the analyzed sample. Most of the examples presented so far require the use of redox probes in the solution, representing a specific drawback for their use in point-of-care systems.

### 2.5. Reproducibility, Repeatability, Specificity, and Stability Studies of Immunosensor Based on Antibody–Ferrocene Conjugates

**Figure 5:**
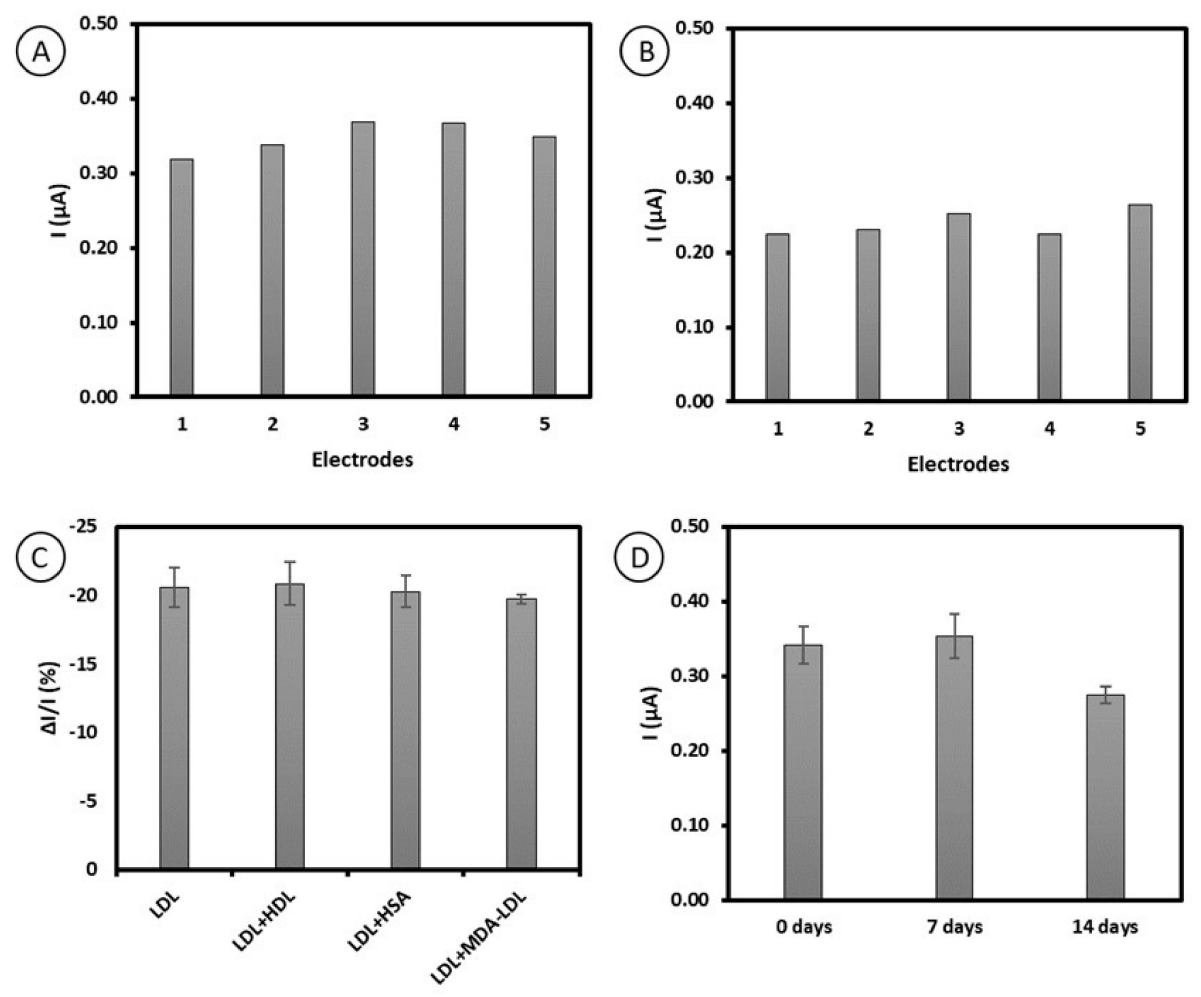
Results of (**A**) reproducibility, (**B**) repeatability, (**C**) selectivity of immunosensor with 0.01 ng/mL LDL with different interferents at a concentration of 0.5 ng/mL (high-density lipoprotein (HDL), human serum albumin (HSA), and malondialdehyde-modified low-density lipoprotein (MDA-LDL)), and (**D**) stability studies registered within 14 days, obtained for the platform based on antibody–ferrocene conjugates.

Reproducibility, repeatability, specificity, and stability studies were conducted in order to evaluate the practicability of the immunosensor based on antibody–ferrocene conjugates. Adequate reproducibility of the platform proposed was observed, proved by relative standard deviation (RSD) values of 6.03% (Figure 5A). Moreover, the RSD values of 7.44% obtained for repeatability studies indicate satisfactory repeatability of the method proposed (Figure 5B). We also evaluated the specificity of the platform based on antibody–ferrocene conjugates in order to express the ability to detect LDL in the presence of possible interferents, namely, human serum albumin, high-density lipoprotein, and malondialdehyde-modified low-density lipoprotein. For this purpose, the immunosensor was used for the detection of 0.01 ng/mL LDL, along with 50-fold higher concentrations of HSA, HDL, and MDA-LDL. As presented in Figure 5C, all tested interferents did not have significant effects on the detection of LDL. Moreover, the relative standard deviations calculated for interferents are in the acceptable range, with 7.7% for HDL, 5.9% for HSA, and 1.5% for MDA-LDL. Thus, the obtained results confirm the high selectivity of the developed immunosensor. The lack of interferences from MDA-LDL is promising. A slight difference in the structure consisting of MDA bound to the positively charged epsilon-amino group of apoB-100 protein lysine residues occurs compared to LDL [40]. Both LDL and MDA-LDL are considered as cardiovascular disease biomarkers, so the selective determination of LDL in the presence of MDA-LDL is important.

Furthermore, the peak current of antibody–ferrocene conjugates immobilized on gold electrodes was monitored for 14 days in order to define the electrode stability. Our results show that the peak current value for Fc retained 102.9% of its initial value, which confirms the stability of the proposed platform for 7 days (Figure 5D).

### 2.6. Application of Immunosensor Based on Antibody–Ferrocene Conjugates in Real Sample Analysis

To assess the suitability of the proposed sensor for testing real samples, LDL was detected in 100-fold-diluted human serum samples. The standard solution of LDL was added to the diluted serum samples, and square wave voltammetry was conducted. The obtained recovery values were in the range between 96.7% and 105.0% and RSD was between 0.38% and 3.50%, demonstrating that the electrochemical platform based on antibody–ferrocene conjugates can reliably detect LDL (Table 2). The results clearly indicate the applicability of this platform in real sample analysis.

## 3. Materials and Methods

### 3.1. Chemicals

Apolipoprotein B monoclonal antibody (AbM-anti-apoB) was acquired from Invitrogen (Legnica, Poland). Magne Protein A beads, 20% slurry, were purchased from Promega (Madison, WI, USA). Ferrocene carboxylic N-hydroxysuccinimide ester (Fc-NHS) was obtained from Fivephoton Biochemicals (San Diego, CA, USA). 4-aminothiophenol (4-ATP), N-hydroxysuccinimide (NHS), N-(3-dimethylaminopropyl)-N-ethylcarbodiimide hydrochloride (EDC), bovine serum albumin (BSA), Na_2_SO_4_, phosphate buffer (PB; sodium phosphate (monobasic, monohydrate), sodium phosphate (dibasic, heptahydrate), pH 7.0), elution buffer (EB; glycine, pH 2.7), neutralization buffer (NB; Trizma base, Trizma hydrochloride, pH 7.5), low-density lipoprotein (LDL), human serum albumin (HSA), and dimethyl sulfoxide (DMSO) were purchased from Sigma-Aldrich (Poznań, Poland). High-density lipoprotein (HDL) was obtained from Merck (Darmstadt, Germany). Malondialdehyde-modified low-density lipoprotein (MDA-LDL) was purchased from Cell Biolabs, Inc. (San Diego, CA, USA). Na_2_SO_4_, H_2_SO_4_, KOH, ethanol, and methanol were purchased from POCH (Gliwice, Poland). Alumina slurries of 0.3 and 0.05 µm were obtained from Buehler (Illinois, IL, USA). All aqueous solutions were prepared based on deionized water (resistivity of 18.2 MΩ cm) with a Milli-Q reagent grade system produced by Millipore (Bedford, MA, USA). Human blood serum was obtained from Sigma-Aldrich (Poznań, Poland).

### 3.2. Equipment

Potentiostat/Galvanostat AutoLab (Methrohm Autolab, The Netherlands) was used for all electrochemical measurements. A three-electrode system with gold as the working electrode, Ag/AgCl as the reference electrode, and Pt wire as the counted electrode was applied for analysis. All three electrodes were manufactured by Bioanalytical Systems (BASi, West Lafayette, IN, USA).

### 3.3. Procedure of Ferrocene-NHS Conjugation to Antibody

Initially, a volume of 50 µL magnetic beads covered with protein A was introduced in the tube and washed twice with 250 µL of 10 mM phosphate buffer, pH 7.0, in order to eliminate storage buffer. Between each washing step and incubation, the beads were collected using a magnetic stand, and after 10 s, the supernatant was removed. Then, 1 mL of AbM-anti-apoB (0.1 mg/mL) prepared in phosphate buffer was added to the tube with beads. The solution was continuously stirred for 1 h at RT. The received AbM-anti-apoB-modified beads were washed twice with PB to reject unbound antibodies. Subsequently, the bound AbM-anti-apoB beads were suspended in 100 µL phosphate buffer. Then, 12 µL Fc-NHS (1 mg/mL prepared in DMSO/H_2_O,1:1) was added to this suspension. The Fc-NHS binding reaction with AbM-anti-apoB proceeded with gentle continuous stirring for 2 h at RT. Thereafter, the unbound ferrocene molecules were discarded through double washing with PB. Afterwards, elution was performed to recover the ferrocene-conjugated antibodies. For this purpose, 100 mL of 50 mM elusion buffer, pH 2.8, was added to the AbM-anti-apoB-Fc-beads and mixed for 5 min at RT. A magnetic stand was used to separate magnetic beads from AbM-anti-apoB-Fc. Eluted samples were placed in the new tube containing neutralization buffer, pH 7.5. The concentration of AbM-anti-apoB-Fc was determined with the Nanodrop Spectrophotometer 2000 (Thermo Scientific, Waltham, MA, USA). The recovery of ferrocene-conjugated antibodies was estimated as 75%, which is in agreement with the literature [23,24]. After washing, the resulting sample was aliquoted and stored at 4 °C until use.

### 3.4. Preparation of Immunosensor Platform Based on Conjugates of Antibodies with Ferrocene

The procedure of cleaning the gold electrode surface was carried out following previously reported protocol [31]. After careful cleaning, the Au electrodes were dipped in an ethanolic solution of 1 mM 4-ATP overnight. Afterwards, to clear out unbound molecules, the electrodes were rinsed with ethanol and Milli-Q H_2_O. Next, to activate the –COOH group at AbM-anti-apoB-Fc, the aqueous solution containing this antibody at a concentration of 0.05 ng/mL and EDC/NHS was incubated for 15 min. Then, 10 µL of AbM-anti-apoB-Fc was dropped onto the electrode surface for 2 h at RT. After this time, the electrodes were rinsed with buffer containing 0.1 M sodium phosphate buffer and 0.15 M Na_2_SO_4_, pH 7.0. Next, 10 µL of 0.01% BSA was deposited onto the gold electrodes for 1 h and then rinsed as before with buffer. These electrodes were subsequently left overnight at 4 °C in the buffer (0.1 M sodium phosphate buffer, 0.15 M Na_2_SO_4_, pH 7.0).

### 3.5. Electrochemical Measurements of LDL

After overnight conditioning, the immunosensor was ready for electrochemical measurements. First, 10 µL of the sample solution containing particular concentrations of LDL in buffer (0.1 M sodium phosphate buffer and 0.15 M Na_2_SO_4_, pH 7.0) was dropped onto the surface of modified gold electrodes. After 30 min of interaction between LDL and AbM-anti-apoB-Fc, electrodes were loaded in the buffer and square wave voltammograms were recorded in the range of -0.1 V to 0.6 V with 10 Hz frequency. In order to detect LDL in human serum samples, the human blood serum samples were filtered with a Millipore AmiconUltracelYM-3, MWCO 3kD, and centrifuged for 60 min at 10,000 RCF. The filtered human serum samples were 100-fold diluted with buffer and used for further testing.

## 4. Conclusions

An immunosensor for the sensitive and selective electrochemical detection of LDL based on antibody–ferrocene conjugates is presented in this work. We propose a one-step electrochemical detection platform based on antibody–ferrocene conjugates. These conjugates perform a dual function of LDL capturing and signaling at the same time. Square wave voltammetry was applied as a sensing technique. The changes in the redox current of ferrocene attached to antibodies, generated by the interfacial reaction between LDL and ferrocene–antibodies conjugates, were employed to quantify the amount of LDL. This immunocomplex formation led to a decrease in the ferrocene current due to the increased spatial blocking. The detection limit recorded by the presented immunosensor was equal to 0.53 ng/mL, which is superior to other immunosensors. An additional advantage of this immunosensor is its high specificity towards typical interferents, namely, human serum albumin, high-density lipoprotein, and malondialdehyde-modified lipoprotein. Moreover, the recovery tests demonstrated the practical applicability of the sensor for the analysis of LDL in human serum samples.

## Figures and Tables

**Table 1:** Comparison of various reported immunosensors for the detection of LDL with the immunosensor proposed in this work based on antibody–ferrocene conjugates.

Substrate	Redox	Electrochemical Method	Limit of Detection ng/mL	Ref.
AuNPs-AgCl@PANI	in solution: [Fe(CN)_6_]^3−/4−^	EIS	0.34 × 10^−3^	[15]
CNT−NiO	in solution [Fe(CN)_6_]^3−/4−^	EIS	6.3 × 10^3^	[20]
CNT-CH/ITO	in solution [Fe(CN)_6_]^3−/4−^	EIS	1.25 × 10^5^	[18]
CysCdS-nNiO/ITO	in solution [Fe(CN)_6_]^3−/4−^	CV	500	[19]
NH_2_-rGO/ITO	in solution [Fe(CN)_6_]^3−/4−^	EIS	5.0 × 10^4^	[17]
PANI–SA LB	in solution [Fe(CN)_6_]^3−/4−^	EIS	-	[16]
Au/4-ATP/AbM/BSA	in solution: [Fe(CN)_6_]^3−/4−^	SWV	0.31	[31]
NiO	on surface NiO	DPV	52.5 × 10^3^	[22,23]
Au/4-ATP/AbM-Fc/BSA	on surface ferrocene conjugates	SWV	0.53	Present work

AuNPs, Au nanoparticles; AgCl@PANI, silver chloride@polyaniline; CysCdS-nNiO, L-cysteine-capped cadmium sulfide quantum dots bound to nickel oxide nanorods; ITO, indium tin oxide; CNT, carbon nanotubes; rGO, reduced graphene oxide; CH, chitosan; NH_2_-rGO, aminated reduced graphene oxide; 4-ATP, 4-aminothiophenol; AbM-Fc, ferrocene monoclonal antibody conjugate; BSA, bovine serum albumin; EIS, electrochemical impedance spectroscopy; SWV, square wave voltammetry; CV, cyclic voltammetry; DPV, differential pulse voltammetry.

**Table 2:** Results of LDL detection in human serum samples.

Spiked C_LDL_ (ng/mL)	Measured C_LDL_ (ng/mL)	Recovery (%)	RSD (%, n = 3)
0.01	0.0105	105.0	3.50
0.02	0.0195	97.5	2.02
0.03	0.0290	96.7	0.38

## Data Availability

The data presented in this study are available on request from the corresponding author.

## Sample Availability

Samples of the AbM-anti-apo-Fc compounds are not available from the authors.
